# Prevention of tau seeding and propagation by immunotherapy with a central tau epitope antibody

**DOI:** 10.1093/brain/awz100

**Published:** 2019-04-30

**Authors:** Marie Albert, Georges Mairet-Coello, Clément Danis, Sarah Lieger, Raphaëlle Caillierez, Sébastien Carrier, Emilie Skrobala, Isabelle Landrieu, Anne Michel, Mathieu Schmitt, Martin Citron, Patrick Downey, Jean-Philippe Courade, Luc Buée, Morvane Colin

**Affiliations:** 1 Inserm, UMR-S 1172, Alzheimer and Tauopathies, Place de Verdun, Lille, France; 2 Université de Lille, Lille Neuroscience and Cognition, Faculté de Médecine, Lille, France; 3 UCB Biopharma, Chemin du Forest, Braine l’Alleud, Belgium; 4 CNRS, UMR8576, Lille, France; 5 CHU-Lille, CMRR, Lille, France; 6 CHU-Lille, EA2694, Department of biostatistics, Lille, France

**Keywords:** immunotherapy, Alzheimer’s disease, seeding, propagation

## Abstract

Tauopathies are neurodegenerative diseases characterized by the intraneuronal accumulation of aggregated tau. The staging of this neurodegenerative process is well established for Alzheimer’s disease as well as for other tauopathies. The stereotypical pattern of tau pathology in these diseases is consistent with the hypothesis that the tau protein can spread in a ‘prion-like’ manner. It proposes that extracellular pathological tau species can transmit pathology from cell to cell. Accordingly, by targeting these spreading species with therapeutic antibodies one should be able to slow or halt the progression of tau pathology. To be effective, antibodies should neutralize the pathological species present in Alzheimer’s disease brains and block their cell-to-cell spread. To evaluate both aspects, tau antibody D, which recognizes an epitope in the central region of tau, and was selected for its outstanding ability to block tau seeding in cell based assays, was used in this study. Here, we addressed two fundamental questions: (i) can this anti-tau antibody neutralize the pathological species present in Alzheimer’s disease brains; and (ii) can it block the cell-to-cell spread of tau seeds *in vivo*? First, antibody D effectively prevented the induction of tau pathology in the brains of transgenic mice that had been injected with human Alzheimer’s disease brain extracts, showing that it could effectively neutralize the pathological species present in these extracts. Second, by using K18 P301L tau fibrils to induce pathology, we further demonstrated that antibody D was also capable of blocking the progression of tau pathology to distal brain regions. In contrast, an amino-terminal tau antibody, which was less effective at blocking tau seeding *in vitro* showed less efficacy in reducing Alzheimer’s disease patient tau driven pathology in the transgenic mouse model. We did not address whether the same is true for a spectrum of other amino-terminal antibodies that were tested *in vitro.* These data highlight important differences between tau antibodies and, when taken together with other recently published data, suggest that epitope may be important for function.

## Teaser

Immunotargeting of extracellular tau could slow the prion-like spreading of neurodegeneration. Albert et al. report that antibodies that recognize a central epitope on tau are the most effective at blocking both seeding and propagation of tau pathological species in transgenic mouse models seeded by materials derived from Alzheimer’s disease brains.

## Introduction

The tau protein is considered to be an intracellular neuronal protein involved in microtubule polymerization and stabilization (Weingarten *et al.*, 1975). The adult human brain contains six tau isoforms that derive from a single gene (*MAPT*) by alternative splicing of exons 2, 3 and 10 (Goedert *et al.*, 1989). The hyperphosphorylation and deposition of tau proteins in insoluble aggregates inside neurons is a hallmark of around 20 pathologies termed tauopathies; these include the well-known Alzheimer’s disease (Spillantini *et al.*, 1998; Buée *et al.*, 2000). These pathologies differ by both the content in tau isoforms [three (3R)- or four (4R)-microtubule-binding domains] and the regional distribution of tau aggregates. The spatio-temporal development of these aggregates in the brain has been described in Alzheimer’s disease (Braak and Braak, 1991; Duyckaerts *et al.*, 1997, 2015; Delacourte *et al.*, 1999; Cho *et al.*, 2016; Cope *et al.*, 2018; Hoenig *et al.*, 2018), progressive supranuclear palsy (Verny *et al.*, 1996; Williams *et al.*, 2007) and argyrophilic grain disease (Saito *et al.*, 2004). Emerging evidence suggests that the spread of tau pathology reflects the propagation of abnormal tau species along neuroanatomically connected brain areas (Dujardin *et al.*, 2014*a*; Calafate *et al.*, 2015; Congdon *et al.*, 2016). This propagation could occur in a ‘prion-like’ manner involving transfer of abnormal tau seeds from a donor cell to a recipient cell in which the recruitment of normal tau generates new tau seeds (Clavaguera *et al.*, 2009; de Calignon *et al.*, 2012; for a review see Mudher *et al.*, 2017).

In line with this hypothesis, tau has been identified recently in the extracellular space, supporting a role in cell-to-cell transfer of pathology (Dujardin *et al.*, 2014*b*; Hanger *et al.*, 2014; Croft *et al.*, 2017; for a review see Sotiropoulos *et al.*, 2017). Extracellular tau is now considered to be the key driver in the spread of pathology making immunotherapy an attractive therapeutic approach. Numerous reports support the benefit of tau immunotherapy in various animal models (Asuni *et al.*, 2007; Boutajangout *et al.*, 2011; Chai *et al.*, 2011; Troquier *et al.*, 2012; d’Abramo *et al.*, 2013; Castillo-Carranza *et al.*, 2014, 2015; Collin *et al.*, 2014; Dai *et al.*, 2015, 2017; Umeda *et al.*, 2015). These models have proven to be useful for analysis of cell-autonomous pathological cellular mechanisms associated with development of tau aggregates as they all tested the effect of immunization with tau antibodies in transgenic mice. However, it is difficult to assess the respective roles of cell-autonomous and non-cell-autonomous mechanisms in propagation of tau pathology in such mice. This led to the development of new tauopathy models based on the intracranial injection of extracellular pathological tau species to be close to the non-cell-autonomous mechanisms (Ahmed *et al.*, 2014; Peeraer *et al.*, 2015). To date, only three studies have addressed the immunization paradigm using either recombinant fibrils (Sankaranarayanan *et al.*, 2015) or human phosphorylated tau isolated from Alzheimer’s disease brain tissues (Dai *et al.*, 2018; Vandermeeren *et al.*, 2018).

Recent comparative studies have begun to address which tau antibodies may be the most efficacious at blocking seeding from human Alzheimer’s disease material *in vitro* (Nobuhara *et al.*, 2017; Courade *et al.*, 2018; Vandermeeren *et al.*, 2018). Courade *et al.* (2018) identified a tau central epitope ‘antibody D’ as being the most efficacious *in vitro*, while a previously described N-terminally directed tau ‘antibody A’ showed little activity. Therefore, we wanted to assess if antibody D could first neutralize the pathological species present in Alzheimer’s disease brains, and second, whether it could block cell-to-cell spread *in vivo.* To answer the first question, we induced tau pathology with Alzheimer’s disease brain-derived material in Tg30tau transgenic mice (Schindowski *et al.*, 2006; Leroy *et al.*, 2007). We showed that targeting the mid-domain of tau with antibody D prevents the appearance of neurofibrillary degeneration in the pyramidal neurons of the CA1 layer. Next, we used a slightly modified strategy to address specifically the effect of antibody D on cell-to-cell spread. We injected K18-P301L in hTauP301L transgenic mice (Peeraer *et al.*, 2015); tau fibrils that are not recognized by tau antibody D. We demonstrated in this model that antibody D is capable of blocking the progression of tau pathology to distal brain regions, as the therapeutic antibody cannot bind to the injected material, it unambiguously demonstrates an effect on spread, even though the seed is artificial.

In conclusion, tau antibody D, which recognizes a central epitope on tau, is more efficacious at blocking pathology induced by Alzheimer’s disease brain-derived material *in vivo* than tau antibody A, which recognizes an N-terminal epitope. Furthermore, antibody D was also shown to effectively block cell-to-cell transfer. These data, when considered together with previously published *in vitro* studies, suggest that the choice of tau epitope could be a critical determinant of therapeutic efficacy of tau antibodies.

## Materials and methods

### Animals

The present experimental research was performed with the approval of an ethical committee (agreement APAFIS#2264–2015101320441671 from CEEA75, Lille, France protocol ASYN-IC-PARKINSON-MO from LA1220040 and LA2220363, Brain l’Alleud, Belgium) and follows European guidelines for the use of animals. The animals (males and females) were housed in a temperature-controlled (20–22°C) room maintained on a 12-h day/night cycle with food and water provided *ad libitum* in specific pathogen free (SPR) animal facility (*n =* 5 mice per cage). Animals were allocated to experimental groups by randomization. THY-tau30 (Tg30tau) mice express human 1N4R tau protein with two pathogenic mutations (P301S and G272V) under the control of the neuron-specific Thy1.2 promoter (Schindowski *et al.*, 2006; Leroy *et al.*, 2007). HtauP301L transgenic mice express human 2N4R tau protein with P301L mutation under the Thy1.2 promoter (Terwel *et al.*, 2005), and were provided by reMYND. Wild-type mice from the same genetic background were used as control.

### Antibodies

Antibodies used for immunohistochemistry and biochemical assays and therapeutic antibodies are described in Supplementary Table 1 and in Courade *et al.* (2018).

### Alzheimer’s disease brain samples and preparation

Non-demented control and human Alzheimer’s disease brain cortical extracts (Braak 6, frontal cortex, Brodmann area 10) were obtained from the Lille Neurobank (fulfilling criteria of the French law on biological resources and declared to competent authority under the number DC-2008-642) with donor consent, data protection and ethical committee review. Samples were managed by the CRB/CIC1403 Biobank, BB-0033-00030. To generate human brain homogenates, samples were homogenized on ice with a glass potter in five volumes (w/v) of phosphate-buffered saline (PBS). Sonicated homogenates (h-AD) were centrifuged at 4°C 3000*g* for 5 min and then the supernatant was aliquoted and kept at −80°C until use. PHF (PHF-AD) samples were purified as described previously (Forest *et al.*, 2013) (Supplementary Fig. 4A).

### P301L-K18 fibrils

The P301L-K18 peptide is composed of the four microtubule-binding regions of tau protein with a pro-aggregative and pathogenic P301L mutation found in frontotemporal dementia with parkinsonism-17 (Supplementary Fig. 1A) (Nacharaju *et al.*, 1999). The P301L-K18 fragment was expressed as soluble His-tagged protein in *Escherichia coli* using standard methods. Cell pellets were lysed (10 mM PBS, pH 7.4, 500 mM NaCl, 20 mM imidazole, 1 U/ml benzonase, 0.2 mg/ml lysozyme, 1× protease inhibitor tablet from Sigma), His-tag cleaved by TEV (Tobacco Etch Virus) protease, and then P301L-K18 peptides were purified using Ni-NTA (Qiagen). The final purification step was carried out by gel filtration Superdex® 75 (GE Healthcare) in 10 mM PBS, pH 7.4, 138 mM NaCl. Purified P301L-K18 peptides (66 mM) were incubated with heparin (266 mM) at 37°C for 5 days (10 mM PBS, pH 7.4, 138 mM NaCl). Resulted P301L-K18-fibrils were centrifuged at 100 000*g* for 1 h, suspended in ammonium acetate (11 mM, pH 7.0) and sonicated. The presence of fibrils was confirmed by thioflavin T fluorescence and negative stain electron microscopy (Supplementary Fig. 2). For thioflavin T assay, 50 µg of K18 fibrils were incubated with 50 µM of thioflavin T in buffer containing 10 mM Na^+^ phosphate, 150 mM NaCl, pH 7.0. Results are expressed as fluorescence units. For electron microscopy, negative stain was performed in 2% uranyl acetate.

### Passive immunization

For both *in vivo* experiments, experimenters were blind to the treatments. The poor exposure of IgG to the brain (St-Amour *et al.*, 2013; Abuqayyas *et al.*, 2013) and the literature (Collin *et al.*, 2014; Yanamandra *et al.*, 2017) led us to select an IgG dose of 30 mg/kg. Antibodies (30 mg/kg) were administered intraperitoneally in 1-month-old Tg30tau mice. Animals received two antibody injections, 7 days and 24 h, before the intra-hippocampal injection of h-AD, and then once a week for 1 month before sacrifice (Supplementary Fig. 1B). Four-month-old htau P301L transgenic mice received intraperitoneal administration of antibodies (30 mg/kg) 24 h before intracranial injection of P301L-K18 fibrils, and then once a week for 6 weeks until sacrifice 24 h later (Supplementary Fig. 1C). The brains were collected for further analysis by immunohistochemistry.

### Stereotaxic injections

Stereotaxic injections of h-AD and recombinant P301L-K18 fibrils were performed in the hippocampus of Tg30tau and htauP301L mice. H-AD (2 µl, 5.5 µg/µl), PHF-AD preparation (1 µg) or PBS (2 µl) were injected in the right hippocampus of 1-month-old anaesthetized (ketamine 100 mg/kg, xylazine 10 mg/kg) Tg30tau mice (weight = 15–20 g) (anterior-posterior: −2.5 mm; medial-lateral: −1 mm; dorsal-ventral: −1.8 mm to bregma; Supplementary Fig. 3A). Sonicated P301L-K18 fibrils (1 µl, 5 µg/µl) or PBS (1 µl) were injected in the right hippocampus of 4-month-old anaesthetized (ketamine 50 mg/kg, medetomidine 0.5 mg/kg) htauP301L mice (weight = 20–25 g) at the following coordinates: anterior-posterior: −1.8 mm; medial-lateral: −1.72 mm; dorsal-ventral: −1.8 mm (Supplementary Fig. 3B). The standard injection procedure consisted of the delivery of 2 µl in Tg30tau using a 10 μl Hamilton glass syringe with a fixed needle or 1 µl in P301L transgenic mice using a Hamilton glass syringe with a fixed needle (point style 3, 33 gauges, 8 mm length; Hamilton). After injection at a rate of 0.2 μl per min, the needle was left in place for 2.5 min before removal to prevent any leakage of the injected material. Injections at the correct coordinates in the CA1 hippocampal region in the ipsilateral hemispheres were validated for both models with stereotaxic injection of Evans Blue (Supplementary Fig. 3).

### Tissue processing, immunohistochemistry, immunofluorescence and quantification

#### Tgtau30 *in vivo* model

Five weeks post-injection, Tgtau30 animals were deeply anaesthetized and transcardially perfused with ice-cold 0.9% saline solution and subsequently with 4% paraformaldehyde for 10 min. The brains were immediately removed, fixed overnight in 4% PFA, washed in PBS, placed in 20% sucrose for 24 h and frozen until further use. Free-floating coronal sections (40-µm thickness) were obtained using a cryostat microtome. For immunohistochemistry, brain sections were washed in PBS-0.2% Triton™ X-100 and treated for 30 min with 0.3% H_2_O_2_. Non-specific binding was blocked using ‘Mouse in Mouse’ reagent (1:100 in PBS, Vector Laboratories) for 60 min. Incubation with the primary antibody in PBS-0.2% Triton™ X-100 was performed overnight at 4°C. After several washes, labelling was amplified by incubation with an anti-mouse biotinylated IgG (1:400 in PBS-0.2% Triton™ X-100, Vector) for 60 min followed by the application of the avidin-biotin-HRP complexe (ABC kit, 1:400 in PBS, Vector) prior to addition of diaminobenzidine tetrahydrochloride (DAB, Vector) in Tris-HCl 0.1 mol/l, pH 7.6, containing H_2_O_2_ for visualization. Brain sections were then mounted, air-dried, counterstained for 1 min in 0.5% Cresyl violet solution, washed with 2% acetic acid, dehydrated by passage through a graded series of alcohol (70%, 95%, 100%) and toluene baths, and finally mounted with VectaMount (Vector Laboratories) or Permount™ (Fisher). For blinded quantification, slide imaging was performed by microscopy using a slide scanner (Axioscan Z1-Zeiss) with a 20× objective. Brain sections were analysed using stereology software (Mercator image analysis system; Explora Nova, La Rochelle, France) using hue, saturation, and intensity to distinguish objects in the image field. Thresholds were established manually using identified objects on a set of slides and these segmentation thresholds remained constant throughout the analysis. The CA1 region of the hippocampus was chosen as quantification zone. We selected and quantified the contra and the ipsi CA1 regions at six brain sections covering the entire hippocampus (bregmas −1.7, −2.06, −2.46, −2.80, −3.16, −3.52). Results are presented either as per cent marker occupancy (immunoreactive area normalized to the whole area analysed) or number of AT8-positive cells.

#### HtauP301L *in vivo* model

HtauP301L transgenic mice were anaesthetized 6 weeks plus 24 h post-fibril injection and transcardially perfused with ice-cold 0.9% saline solution containing 10 U/ml heparin for 7 min, and then with ice-cold 4% paraformaldehyde prepared in 0.1 M PBS for 7 min at a flow rate of 6 ml/min. The brains were dissected and post-fixed overnight with the same fixator at 4°C. The brains were washed in cold PBS overnight, washed again in PBS for a minimum of 1 h and transferred to PBS containing 15% sucrose and 0.01% NaN_3_, and stored at 4°C until sectioning. Sectioning and immunohistochemistry were performed by Neuroscience Associates. Free-floating coronal sections (40-µm thickness), obtained using a cryostat microtome, were washed with Tris-buffered saline (TBS) containing 0.3% Triton™ X-100, and treated with 0.9% H_2_O_2_. Non-specific binding was blocked using 1.26% whole normal serum. Incubation with the biotinylated AT8 primary antibody in PBS-0.3% Triton™ X-100 was performed overnight at room temperature. Following rinses in TBS, sections were incubated with an avidin-biotin-HRP complex (Vectastain Elite ABC kit, Vector Laboratories) for 1 h at room temperature. Following rinses, the sections were treated with DAB and 0.0015% H_2_O_2_ to create a visible reaction product, mounted on gelatinized (subbed) glass slides, air-dried, dehydrated in alcohols, cleared in xylene, and coverslipped with Permount™. Whole slide imaging was performed using Axioscan Z1 slide scanner (Zeiss) with a 20× objective. For immunofluorescence, brain sections were permeabilized 15 min in PBS-0.3 % Triton™ X-100 and further incubated 12 h at room temperature with biotin-conjugated AT8 antibody. AT8 labelling was revealed using Alexa Fluor^®^ 546 streptavidin-conjugated (1:1000, 1 h at room temperature). Sections were finally counterstained with 4′,6-diamidino-2-phenylindole (DAPI, 300 nM) and whole slide imaging was performed using slide scanner. Brain sections of htauP301L transgenic mice were analysed with VisioPharm 6 software (VisioPharm) using the linear Bayesian algorithm. For blinded quantification, AT8 signal was quantified in the entire contralateral and ipsilateral hippocampal regions on 20 immunostained cryostat sections from bregma −2.06 to −3.64 mm. Results are reported as per cent marker occupancy corresponding to the ratio of immunoreactive area normalized to the area of the region of interest.

### Extraction of sarkosyl-insoluble tau fraction

Five weeks post-injection, mice were sacrificed by cervical dislocation. Brains were extracted and immediately frozen in 2-methylbutane. CA1 regions were recovered using an acrylic coronal brain matrix (World Precision Instruments). Sarkosyl extraction was performed as described previously (d’Orange *et al.*, 2018). Briefly, 0.23% Triton™ X-100 was added to each sample. The sample was sonicated before centrifugation at 5000 *g* for 10 min at 4°C. The supernatant was collected for ultracentrifugation at 100 000*g* for 1 h at 4°C. The pellet was resuspended in 1% sarkosyl, sonicated and ultracentrifuged at 100 000*g* for 1 h at 4°C. The pellet containing the sarkosyl insoluble fraction was then resuspended in 100 µl of 2× lithium dodecyl sulphate.

### Western blot analysis

Western blotting was performed as described previously (d’Orange *et al.*, 2018). Briefly, samples were loaded onto a 4–12% Bis-Tris NuPAGE^®^ Novex^®^ gel (Invitrogen), followed by transfer onto a 0.45 µm nitrocellulose or polyvinylidene difluoride (PVDF) membrane, using the Novex system from Life Technologies (XCell II™ blot module). The membrane was then incubated either with or without blocking solution for 1 h at room temperature before incubation with primary antibody overnight at 4°C. The membrane was then incubated for 1 h with the appropriate secondary antibody. The signal was visualized using ECL western blotting detection reagents (GE Healthcare). For antibody dilutions and blocking solutions, see Supplementary Table 1.

### Statistical analysis

All experiments were performed blinded coded. Immunohistochemistry data are described as mean ± standard error of the mean (SEM). Normality of distributions was assessed graphically and using the Shapiro-Wilk test. Comparisons were performed using Student *t*-test or Mann-Whitney U-test in case of non-Gaussian distribution. The distribution was evaluated graphically and using the Shapiro-Wilk test for each parameter. The populations were not Gaussian except for one (biochemical assay for M19G antibody). Statistical testing was done at the two-tailed α-level of 0.05. Data were analysed using the SAS software package, release 9.4 (SAS Institute, Cary, NC).

### Data availability

The data that support the findings of this study are available from the corresponding author upon reasonable request.

## Results

### Human Alzheimer’s disease brain homogenate efficiently seeds tau in young Tg30tau mice

To follow human tau seeding *in vivo*, we developed a model consisting of the injection of Alzheimer’s disease brain-derived material into the hippocampus of 1-month-old Tg30tau mice. At this age, tau pathology is very weak (Leroy *et al.*, 2007). First, we compared the capacity to induce tau pathology of purified PHF (PHF-AD) (Davies *et al.*, 2013) and human Alzheimer’s disease brain homogenate (h-AD). H-AD or PHF-AD was injected into the right hippocampus of 1-month-old Tg30tau mice and their respective ability to recruit endogenous human mutated tau was compared by immunohistochemistry using AT8 or AT100 antibodies. Both h-AD and PHF-AD induced the development of tau pathology in the ipsilateral and contralateral side of the hippocampus. AT8 and AT100 immunoreactivity was mainly observed in the cell body of CA1 pyramidal neurons as well as in their apical and dendrites basal processes (Supplementary Fig. 4B–G). To identify the best window of tau pathology development for the assessment of therapeutics in this transgenic mouse models, we tested different quantities of Alzheimer’s disease brain-derived material (1 or 2 µg PHF-AD). H-AD was more efficient than purified PHF-AD (1 or 2 µg) at inducing tau pathology (Supplementary Fig. 4B–G). Thus, we selected h-AD for the following experiments.

To quantify the seeding ability of h-AD in this model, h-AD (Supplementary Fig. 5B and F) or PBS (Supplementary Fig. 5C and G) were injected into the CA1 layer of Tg30tau mice and the appearance of tau pathology was evaluated 5 weeks post-injection by immunohistochemistry. Additionally, to validate the ability of h-AD to recruit and seed human mutated tau in Tg30tau mice, wild-type mice were also injected with h-AD (Supplementary Fig. 5A and E).

The effect of h-AD injection in Tg30 mice on tau hyperphosphorylation was first analysed using AT8 antibody. H-AD injection resulted in a 12-fold increase in tau phosphorylation in the ipsilateral CA1 (Supplementary Fig. 5B and D, *P < *0.0001) and a 5-fold increase in the contralateral side (Supplementary Fig. 5D, *P = *0.0003) compared to animals injected with PBS (Supplementary Fig. 5C and D). We also looked at the effect of h-AD on the development of neurofibrillary tangles using AT100 antibody. H-AD injection resulted in a 18-fold increase in the ipsilateral side (Supplementary Fig. 5F and H, *P < *0.0001) and a 3.8-fold increase in the contralateral hippocampus (Supplementary Fig. 5H, *P = *0.0136) compared to animals injected with PBS (Supplementary Fig. 5G and H). Thus, intracranial injection of h-AD in the CA1 layer of 1-month-old Tg30tau mice induces, 5 weeks post-injection, the appearance of neurofibrillary tangles in pyramidal neurons of the CA1 layer. Furthermore, no AT8 and very low AT100 immunoreactivity, corresponding to the background signal, was detected in littermate wild-type mice injected with h-AD, similar to Tg30tau mice injected with PBS (for AT8 see Supplementary Fig. 5A, C and D, ipsilateral side, *P = *0.0907 and contralateral side, *P = *0.105; for AT100 see Supplementary Fig. 5E, G and H, ipsilateral side, *P = *0.0277 and contralateral side, *P = *0.0005). These results indicate that human brain-derived material recruits and seeds the human mutated tau overexpressed in Tg30tau mice into neurofibrillary tangles.

### Immunization with a mid-region epitope tau antibody reduces human tau seeding

Two murine anti-tau antibodies (IgG gamma 1), named A and D, recognizing the 50–65 kD PHF triplet banding pattern, which is classical in Alzheimer’s disease (Courade *et al.*, 2018) and an isotype antibody recognizing the human TNFα but not murine TNFα (negative control antibody) were used for immunization study. The murinized antibody A is a previously-published antibody binding to the amino-terminal domain of tau (amino acids 15–24, WO 2014/028777A2, Supplementary Fig. 1A). Antibody D binds to a central epitope located just before the four microtubules binding domains of tau (amino acids 235–250, Courade *et al.*, 2018, Supplementary Fig. 1A). Antibody D blocked seeding induced by Alzheimer’s disease and progressive supranuclear palsy seeds, while antibody A was not very efficient in the cell-based assay (Courade *et al.*, 2018). Furthermore, while both antibodies A and D bind to PHF-AD tau, they had different extrinsic affinities, with K_D_ values of 0.12 nM for antibody A and 0.8 nM for antibody D (Supplementary Table 1B).

**Figure 1:**
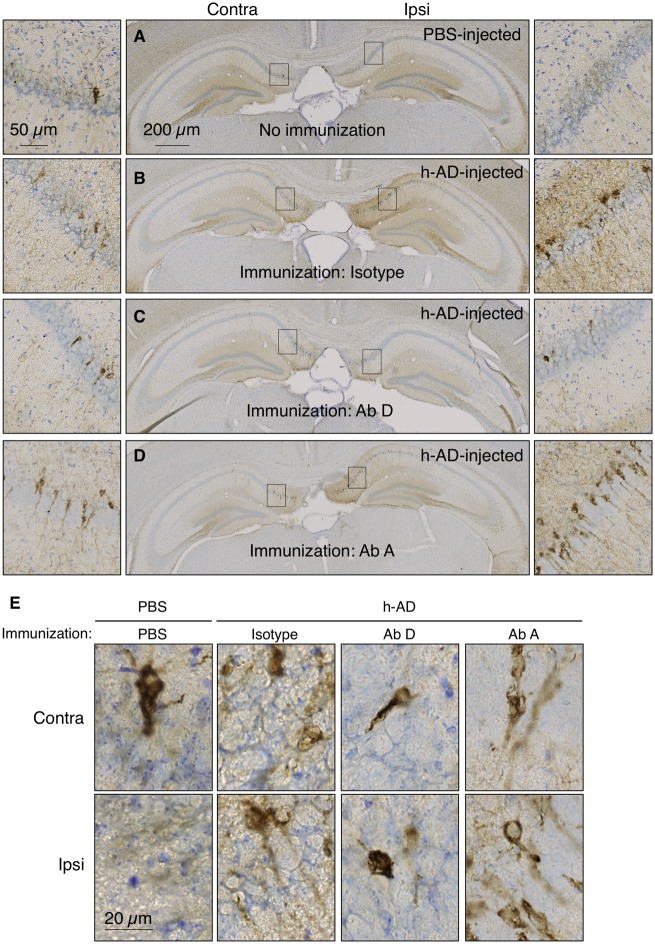

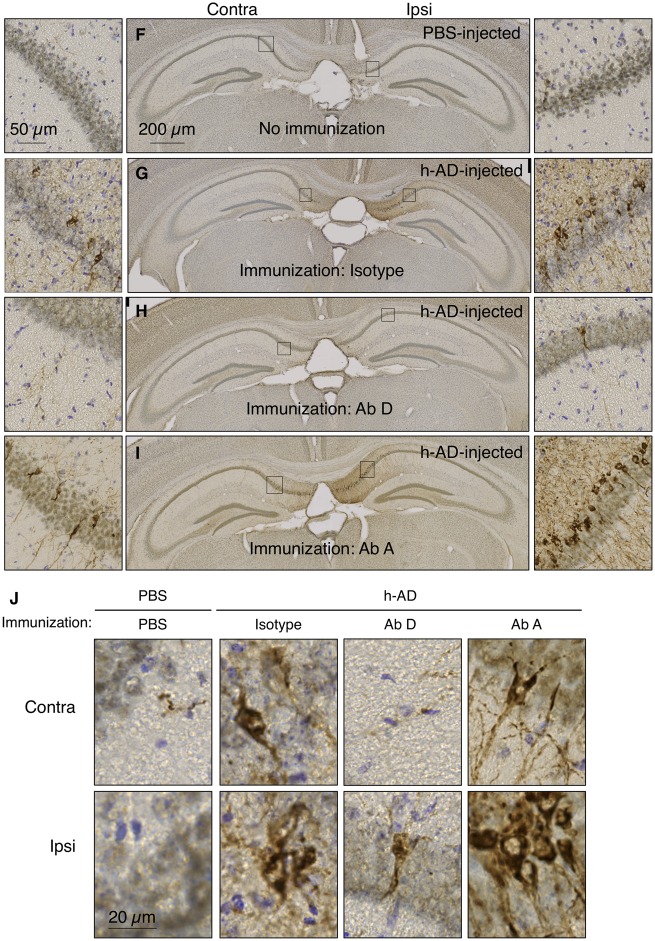
**Anti-tau immunization prevents human tau seeding induced by extracellular human pathological tau species.** One-month-old Tg30tau mice were treated with intraperitoeal injections (30 mg/kg) of antibodies A (**D** and **I**), D (**C** and** H**) or isotype (**B** and **G**) 1 week and 24 h before stereotaxic injection of h-AD (**B**–**D** and** G**–**I**) in the right CA1 layer. Mice that received stereotaxic injection of PBS instead of h-AD were used as negative control (**A** and **F**). Antibodies or PBS were then administered intraperitoneally once a week for 1 month. Mice were sacrificed 5 weeks post-injection and the whole brains were processed for immunohistochemical analysis using AT8 (**A**–**D**) or AT100 antibodies (**F**–**I**). Sections from the hippocampus (injection site) are shown (**A**–**D** and **F**–**I**). Enlargements of CA1 layers (black squares) are shown on the left and right part of hippocampus sections. (**E** and **J**) Higher magnifications at the cell level were shown. Ab = antibody.

**Figure 2:**
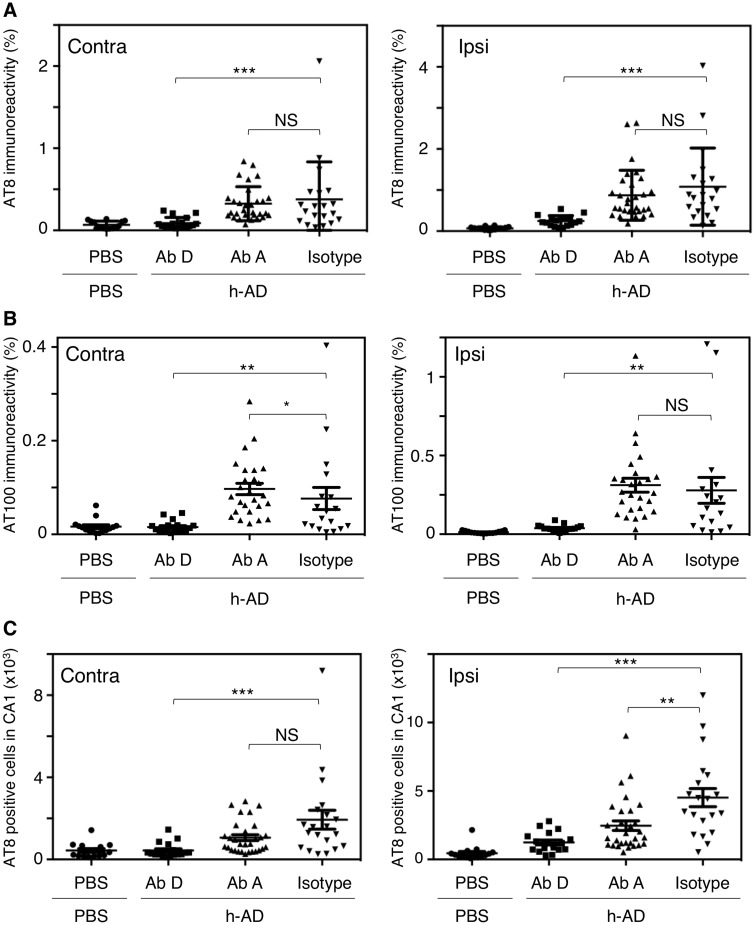
**Quantification of tau pathology after anti-tau immunization in the seeding tg30tau model. **(**A**) Percentage of AT8 immunopositive areas (**A**) in the ipsi- and contralateral CA1 layer. Statistical values: antibody D versus isotype, in the ipsilateral side: Z = −4.22, *P < *0.0001 and in the contralateral side: Z = −3.55, *P = *0.0004; antibody A versus isotype, in the ipsilateral side: Z = 0.7, *P = *0.482 and in the contralateral side: Z = −0.62, *P = *0.535). (**B**) Percentage of AT100 immunopositive areas in the ipsi- and contralateral CA1 layer. Statistical values: antibody D versus isotype, in the ipsilateral side: Z = −3.27, *P = *0.0011 and in the contralateral side: Z = −2.7, *P = *0.0068; antibody A versus isotype, in the ipsilateral side: Z = −1.76, *P = *0.0784 and in the contralateral side: Z = −2.25, *P = *0.0243). (**C**) Number of AT8-positive cells in the CA1 layer Statistical values: antibody D versus isotype, in the ipsilateral side: Z = −4.16, *P < *0.0001 and in the contralateral side: Z = −4.02, *P < *0.0001; antibody A versus isotype, in the ipsilateral side: Z = 2.84, *P = *0.0045 and in the contralateral side: Z = 1.61, *P = *0.105). For **A**–**C** six brain sections covering the entire hippocampus were quantified (Bregma −1.7 to −3.52). Data are presented as mean ± SEM and analysed by Mann-Whitney U-tests (*n* = 15 Tg30tau mice + PBS, *n = *18 Tg30tau mice + h-AD + antibody D, *n = *23 Tg30tau mice + h-AD + antibody A, *n = *18 Tg30tau mice + h-AD + isotype). *P < 0.05, **P < 0.01. Ab = antibody.

Passive immunization was initiated in Tg30tau mice injected in the right hippocampus with h-AD (Supplementary Fig. 1B) and brains were processed for immunohistochemistry using AT8 and AT100 antibodies to evaluate tau lesions. In our experimental design, two control groups were added: (i) a negative control group in which 1-month-old Tg30tau mice received intracranial injection of PBS and intraperitoneal injections of PBS rather than antibodies. This group defines the basal level of tau pathology that could develop in this transgenic model in the absence of tau seeds; and (ii) a positive control group in which 1-month-old Tg30tau mice received intracranial injection of h-AD and intraperitoneal injections of isotype control antibody. This group defines the pathology mediated by the h-AD (Supplementary Fig. 1B). In the negative control group, AT8 was detected at very low levels, while AT100 was undetectable in the ipsi- and contralateral CA1 (Fig. 1A and F, respectively). In h-AD injected Tg30tau mice treated with the control isotype antibody, both AT8 and AT100 immuno-positive neurons were found in the CA1 fields of the hippocampus, with stronger staining in the ipsi- than in the contralateral side (Fig. 1B and G, respectively). Antibody D treatment strongly reduced AT8 (Figs 1C and 2A) and AT100 immunoreactivity (Fig. 1H and 2B) in the ipsilateral CA1 compared to control isotype antibody treatment. In contrast, antibody A immunization had no effect on AT8 immunoreactivity (Figs 1D and 2A); although a decrease in the number of AT8-positive cells was observed, this was, however, less than that seen with antibody D (Fig. 2C).

**Figure 3:**
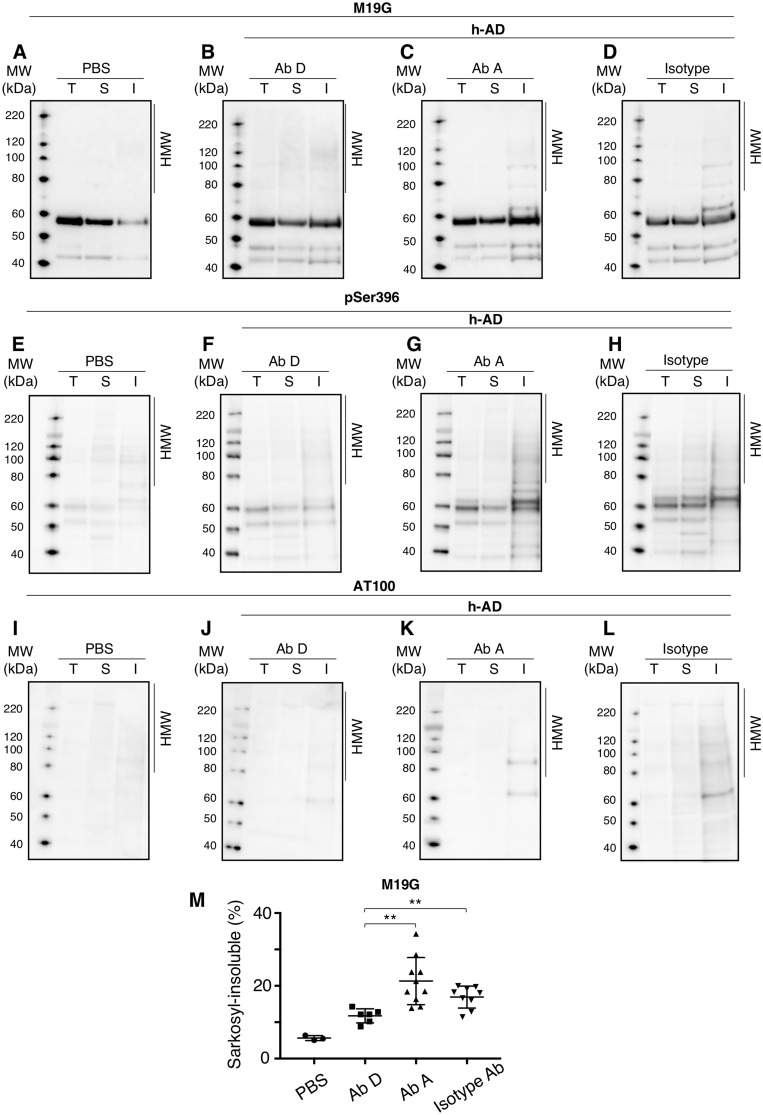
**Biochemical characterization reveals drastic reduction of insoluble and high molecular species after tau passive immunization with antibody D.** One-month-old Tg30tau mice were treated with intraperitoneal injections (30 mg/kg) of antibody A (*n = *10) (**C, G** and** K**), antibody D (*n = *6) (**B, F** and** J**) or isotype antibodies (*n = *9) (**D, H** and** L**) 1 week and 24 h before stereotaxic injection of h-AD (**B**–**D**, **F**–**H** and **J**–**L**) or PBS (*n = *3) (**A**,** E** and** I**) in the right CA1 layer. Antibodies or PBS were then administered intraperitoneally once a week for 1 month. Mice were sacrificed 5 weeks post-injection and the whole brains were processed for western blot after sarkosyl soluble and insoluble extractions using M19G (**A**–**D**), pSer396 (**E**–**H**) or AT100 (**I**–**L**) antibodies. I = sarkosyl-insoluble fraction; S = sarkosyl-soluble fraction; T = total homogenate. (**M**) M19 immunoreactivity quantifications of the sarkosyl-insoluble fraction. Unpaired Student *t*-tests were applied (*n = *10 Tg30tau mice + h-AD + antibody A, *n = *6 Tg30tau mice + h-AD + antibody D, *n = *9 Tg30tau mice + h-AD + isotype); antibody D versus isotype; *t* = −3.68, *P = *0.0028, antibody D versus antibody A; *t* = 4.35, *P = *0.0011. **P < 0.01. Ab = antibody.

These results suggested that immunization with antibody D prevented the formation of neurofibrillary tangles induced by injection of extracellular h-AD. Reduction in immunoreactivities did not result from steric hindrance of AT8 and AT100 with antibody D since pre-incubation of htauP301L hippocampal slices with antibody D (40 µg/ml) did not affect AT8 and AT100 immunoreactivities (Supplementary Fig. 6A and B, respectively). Passive immunization efficacy was further supported using biochemical approaches. The nature of tau aggregates present in the CA1 layer of the ipsilateral hippocampus was assessed by soluble and insoluble-sarkosyl extraction. CA1 homogenates, sarkosyl-soluble and sarkosyl-insoluble extracts were analysed by western blots using antibodies recognizing N-terminal tau (Fig. 3A–D, M19G) and pathologically relevant phosphorylation tau epitopes (Fig. 3E–H, anti-pSer396 and Fig. 3I–L, AT100). As expected, sarkosyl-insoluble materials were weakly identified in the PBS-injected group. Conversely, in hAD-injected groups, sarkosyl-insoluble materials characterized by high molecular weight species, pS396 and AT100-immunoreactivities, were higher in control isotype and antibody A-treated mice than in the antibody D-treated mice. High molecular weight species were mainly labelled by pSer396 and AT100 antibodies, which recognize epitopes mostly found in PHF-like structures (Buée-Scherrer *et al.*, 1996; Allen *et al.*, 2002; Augustinack *et al.*, 2002). Antibody D strongly reduced the level of sarkosyl-insoluble material detected by M19G antibody (highly aggregated tau species) compared to the control isotype antibody (Fig. 3M; 1.6-fold, *P = *0.0028) and compared to antibody A groups (Fig. 3M; 2-fold, *P = *0.0011).

To investigate the ability of tau immunization not only to prevent tau seeding but also its propagation, we analysed AT8 and AT100 immunoreactivities in the contralateral CA1 field. Antibody D treatment strongly reduced AT8 (Figs 1C and 2A) and AT100 immunoreactivity (Figs 1H and 2B) compared to control isotype antibody treatment. Conversely, antibody A immunization had no marked protective effect, neither on AT8 immunoreactivity or AT100 immunoreactivity (Figs 1D and 2A), nor on AT8-positive cell number (Fig. 2C).

Together, these results strongly suggest that antibody D did not only prevent the seeding of host endogenous and soluble human tau in the ipsilateral CA1 but also prevent the propagation of pathological tau species from the ipsi- to the contralateral CA1. However, diffusion of the injected material from the ipsi- to the contralateral side during the injection procedure might bias this conclusion (Supplementary Fig. 3). Antibody D recognized h-AD (Courade *et al.*, 2018) and might thus prevent its diffusion and uptake in the contralateral CA1.

### P301L-K18 fibrils seeded tau in young htauP301L transgenic mice

To determine the ability of antibody D to block the propagation of extracellular and pathological tau species, recombinant P301L-K18 fibrils, not recognized by antibody D, were injected into the right hippocampus of 4-month-old htauP301L transgenic mice. Six weeks post-injection, htauP301L transgenic mouse hippocampi were processed to analyse AT8-immunoreactivity (Supplementary Fig. 7). In P301L-K18 fibrils-injected htauP301L transgenic mice, AT8-immunoreactivity was higher than in PBS-injected htauP301L transgenic mice. AT8 immunoreactivity was lower in the contralateral (Supplementary Fig. 7A and C, 23.5-fold) compared to the ipsilateral (Supplementary Fig. 7C, 67-fold) hippocampus, after injection of P301L-K18 fibrils compared to PBS (Supplementary Fig. 7A and B, *P = *0.0004). AT8 immunoreactivity was not associated with the injected P301L-K18 fibrils as its epitope is localized outside the microtubule-binding regions (Supplementary Fig. 1A). These results indicated that extracellular P301L-K18 fibrils induced seeding of the host human endogenous mutated P301L tau inside neurons.

### Immunization with antibody D strongly prevented human tau propagation

**Figure 4:**
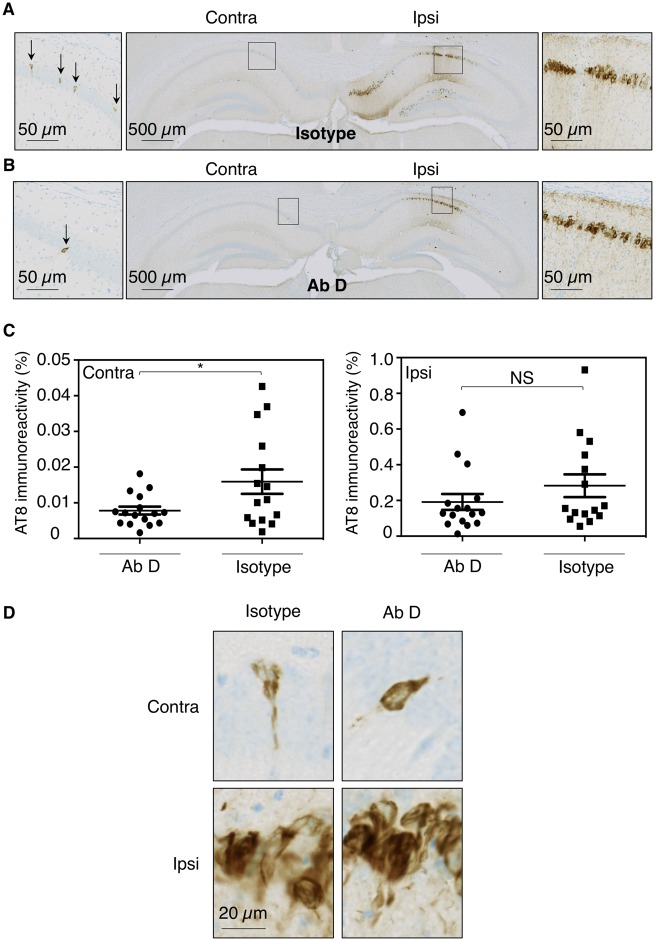
**Anti-tau immunization prevents tau spreading induced by extracellular pathological tau species. **Four-month-old htauP301L transgenic mice were treated with intraperitoneal injections (30 mg/kg) of isotype control antibodies (**A**) or antibody D (**B**) 24 h before stereotaxic injection of P301L-K18 fibrils in the right hippocampus. Antibodies were then administered intraperitoneally once a week. Mice were sacrificed 6 weeks post-injection plus 24 h, and the whole brains were processed for immunohistochemical analysis using AT8 antibody. Sections from the hippocampus (injection bregma) are shown (**A** and **B**). Enlargements of CA1 layers (boxed areas) are shown on the *left* and *right* of hippocampus sections. Higher magnifications at the cell level were shown in **D**. Percentage of AT8 immunopositive areas in the ipsi- and contralateral hippocampus (**C**, antibody D versus isotype, in the ipsilateral side: Z = 1.64, *P = *0.109 and in the contralateral side: Z = 2.19, *P = *0.0282). Twenty brain sections (bregma −2.06. to −3.64 mm) were quantified and data are presented as mean ± SEM and analysed by Mann-Whitney U-tests (*n = *16 hP301L transgenic mice+P301L-K18+antibody D, *n = *15 hP301L transgenic mice+P301L-K18+isotype). *P < 0.05.

The model described above was used to test the effect of passive anti-tau immunotherapy on tau spreading. The immunization process was initiated in 4-month-old htauP301L transgenic mice with repeated intraperiotoneal injections of antibody D or control isotype antibodies (Supplementary Fig. 1C). Mice were sacrificed and the brains were processed for immunohistochemistry using AT8 antibody (Fig. 4). Following control isotype antibody immunization, AT8 immunoreactivity was detected in both ipsi- (Fig. 4A, right) and contralateral (Fig. 4A, left) CA1 fields. AT8 immunoreactivity was observed in the cell body and processes of hippocampal neurons (Fig. 4D). As expected, immunization with the antibody D did not reduce AT8-immunoreactivity in the CA1 ipsilateral to the fibril injection (Fig. 4C, *P = *0.1009), as the epitope of the antibody is not present in the fibrils and it therefore cannot inhibit its uptake. However, as would be expected for an antibody having an effect on spread, it reduced AT8 immunoreactivity in the contralateral hippocampus (Fig. 4B and C, 2-fold, *P = *0.00282). Knowing that the antibody D does not bind P301L-K18 fibrils and is not able to block their uptake into neurons, reduction in AT8-immunoreactivity in the contralateral hippocampus indicated that the antibody D prevented tau propagation mediated by tau species secreted by neurons of the ipsilateral hippocampus to neurons in the contralateral hippocampus.

Together, these results showed that the antibody D, directed to the mid-region of tau (amino acids 235–250), was efficient at preventing both seeding and propagation.

## Discussion

The idea that tau pathology may spread in a ‘prion-like’ manner was first proposed some time ago (Alonso *et al.*, 1996; Su *et al.*, 1997). More recently, work from the Tolnay and Goedert groups showed for the first time the *in vivo* induction and propagation of tau pathology using transgenic mouse lines (Clavaguera *et al.*, 2009). Injection of brain homogenates from P301S tau mutant transgenic mice into human wild-type tau transgenic mice brain induced tau pathology in mice which would not otherwise have developed tau pathology. This pathology progressed over time to both neighbouring and distant synaptically connected brain neurons (Clavaguera *et al.*, 2009). The spreading hypothesis states that an extracellular pathological species secreted into the interstitial fluid is taken up by a neighbouring cell, probably through the neural network (Braak and Del Tredici 2011). Although this process has not been fully demonstrated in humans in a pathological context (Braak and Braak, 1991; Duyckaerts *et al.*, 1997, 2015; Delacourte *et al.*, 1999; Cho *et al.*, 2016; Cope *et al.*, 2018; Hoenig *et al.*, 2018), numerous studies demonstrate its existence *in vitro* and *in vivo* in murine models (for a review see Mudher *et al.*, 2017). From a therapeutic point of view, the postulated presence of an extracellular pathological species has generated much hope for using active and passive immunization strategies to counter the progression of tau pathology. Reducing or stopping the spread of tauopathies requires taking into account the intercellular propagation of pathological species but also their seeding abilities. Since the early 2000s, models have been set up to investigate therapeutic antibodies but few have directly studied extracellular species (for a review see Pedersen and Sigurdsson, 2015).

Recently, we have demonstrated *in vitro* that the choice of epitope determines efficacy of therapeutic anti-tau antibodies in a functional assay with human Alzheimer tau (Courade *et al.*, 2018). In this study, the ability of various tau antibodies to neutralize pathology induced by human pathological seeds was determined in a robust and quantifiable cell-based assay. The tau central epitope antibody D (amino acids 235–250) was most efficacious, while a previously described N-terminally directed tau antibody A (amino acids 15–24) showed little activity despite exhibiting high affinity to both monomeric tau and PHF (Supplementary Table 1 and Courade *et al.*, 2018).

In the current study we aim to follow-up on this *in vitro* work *in vivo* and to address two fundamental questions, can antibody D neutralize the pathological species present in Alzheimer’s disease brain and can it also block cell to cell spread *in vivo*? Additionally, we compared the *in vivo* efficacy of antibodies D and A to assess whether the differential activity observed on human Alzheimer’s disease seeds *in vitro* translates *in vivo.* Specific mouse models were chosen to evaluate the ability of these therapeutic tools to block the seeding or the propagation of pathological tau species.

The first model is based on the injection of Alzheimer’s disease brain derived material to validate the ability of antibody D to prevent the seeding of human pathological species. A limitation of such studies is that the precise nature of the spreading species present in the interstitial fluid of tauopathy patients is not yet known and is probably different from those we injected in our models, which contain both intra- and extracellular materials. The induction of tau pathology is likely to be modulated by the nature of extracellular tau species. For instance, we show here for the first time that the injection of Alzheimer’s disease homogenate in the brain of transgenic mice overexpressing a mutated human tau protein (Tg30tau) has a stronger seeding capability than the injection of PHF preparations to seed endogenous human tau.

Previously, we have demonstrated that antibody D was much more efficacious than antibody A at preventing seeding induced by human brain extracts in a cell-based assay. Here we show that these results translate in our *in vivo* model where we induced tau pathology by injecting Alzheimer’s disease patient-derived homogenates directly into the brain of young Tg30tau mice. At the time of injection, these mice had no discernible endogenous tau pathology, which allowed us to evaluate the effectiveness of the antibodies at reducing pathology induced by the injection of human Alzheimer’s disease lysates. Antibody D significantly reduced neurofibrillary tangles, quantified by AT100 antibody both by histochemical and biochemical approaches. Importantly, while the terminal plasma levels of antibody A are slightly lower than those of antibody D, they are not dramatically different. Antibody D is ∼1.6-fold higher, but a small increase in the level of this antibody should (all things being equal) be more than compensated for by the fact that antibody A has about a 7-fold higher affinity for PHF-AD. On balance, we do not believe that this minor difference in exposure is sufficient to explain the total absence of efficacy of antibody A in the Alzheimer’s disease intracerebral injection model. In absence of tau immunodepleted human brain extract, we speculated that this reduction in tau seeding is resulting from the targeting of human tau species by antibodies. This is largely supported by literature showing that immunodepleted brain extracts (i) are no more able to induce tau pathology when injected in the brain (Clavaguera *et al.*, 2009); and (ii) prevent antibodies effects (Nobuhara *et al.*, 2017; Vandermeeren *et al.*, 2018).

Although this model allowed us to validate the efficacy of antibody D *in vivo*, it did not prove that it was capable of blocking the spread of tau pathology, as the antibody may directly bind tau species present in the injected lysate; meaning it might block the uptake of the seeds into the neurons and/or the spreading. Additionally, injected material in one hemisphere could diffuse to the contralateral side. The effect observed with the antibody D in the contralateral hemisphere cannot therefore be interpreted with certainty in terms of an effect on propagation, hence the need to evaluate this property in a second more mechanistic model. The use of fibrillar material that does not contain the epitope recognized by antibody D (recombinant P301L-K18 fibres) allowed us to address the issue of propagation specifically. Just like the Alzheimer’s disease lysate, these fibrils were also capable of inducing tau pathology after intracranial injection. As expected antibody D did not reduce pathology at the site of injection as it cannot block the uptake of K18 fibrils. In contrast, antibody D significantly reduced AT8-immunoreactivity in the contralateral hemisphere. Importantly, as the AT8 epitope is also located outside of the P301L-K18 fibrils, AT8 antibody is also unable to directly recognize the recombinant injected fibrils. So the observed AT8 signal cannot be due to the injected material but rather due to the phosphorylation of endogenous tau. Together, these data lead to the conclusion that antibody D is really preventing the propagation of human tau species from the ipsilateral to the contralateral side and their subsequent ability to seed human tau species on the contralateral side. The way it is preventing tau spreading is not defined here but the clearance of pathological tau species might imply neuronal (prevention of trans-synaptic tau transfer) as well as non-neuronal routes such as glial and glymphatic pathways (Iliff *et al.*, 2014; for a review see Jadhav *et al.*, 2019).

In this study, we thus provide evidence in two murine models that antibody D is able to block cell-to-cell spread *in vivo* and to effectively neutralize the pathological species present in human Alzheimer’s disease brains.

Finally, tau antibody A, which targets the amino-terminal of the tau protein, was less efficacious in the Alzheimer’s disease seeding model than the central epitope tau antibody D, suggesting that as has been demonstrated in cellular systems, targeting the right tau epitope may also be important for *in vivo* efficacy. As humanized versions of both antibodies A and D are currently in clinical development, these data should be taken into account when evaluating efficacy readouts from ongoing clinical trials, especially as most of the antibodies currently in clinical development target the N-terminal part of tau, often with high affinity. Many studies have shown that the tau protein can be cleaved at both the amino- and carboxy-terminal ends leading to the formation of truncated tau species. The biochemical analysis of Alzheimer’s disease patient brains also suggests the existence of cleaved forms (Wischik *et al.*, 1988; Watanabe *et al.*, 1999; Zilka *et al.*, 2012; Derisbourg *et al.*, 2015) and finally, a mass spectrometric analysis of tau from CSF of Alzheimer’s disease patients identified multiple fragments (Barthélemy *et al.*, 2016*a*, *b*; Sato *et al.*, 2018). The epitope of antibody A and other amino terminal antibodies may therefore be less accessible due to cleavage or masked during the folding of the amino-terminal part of the tau protein during the progression of pathology (Jicha *et al.*, 1997). However, it remains to be seen how these findings will translate to the intact human brain; the brain lysates used in our assays may not fully resemble the seeds that spread Alzheimer’s pathology. Finally, some amino-terminal truncation of tau seen in the post-mortem brain may occur after death and thus, some amino-terminal antibodies could be more effective in patients than in these preclinical assays.

Altogether, the *in vivo* results we provide in this study confirm the recently generated data on the characterization of different antibodies, including antibodies A and D, and their ability to prevent seeding in a cell-based assay. Our work emphasizes the need to test different antibodies in different experimental models to select the best candidate for clinical trials.

## Supplementary Material

awz100_Supplementary_Material

## Abbreviations

h-AD: human Alzheimer’s disease brain homogenate
PHF: paired helical filament
