# Supplementary material for: Prevention of tau seeding and propagation by immunotherapy with a central tau epitope antibody
Source: Brain. 2019 Apr 30;142(6):1736–50. doi: 10.1093/brain/awz100 (PMC6536853; doi:10.1093/brain/awz100)
Supplement: awz100_Supplementary_Material [file awz100_supplementary_material.pdf]

Supplementary material

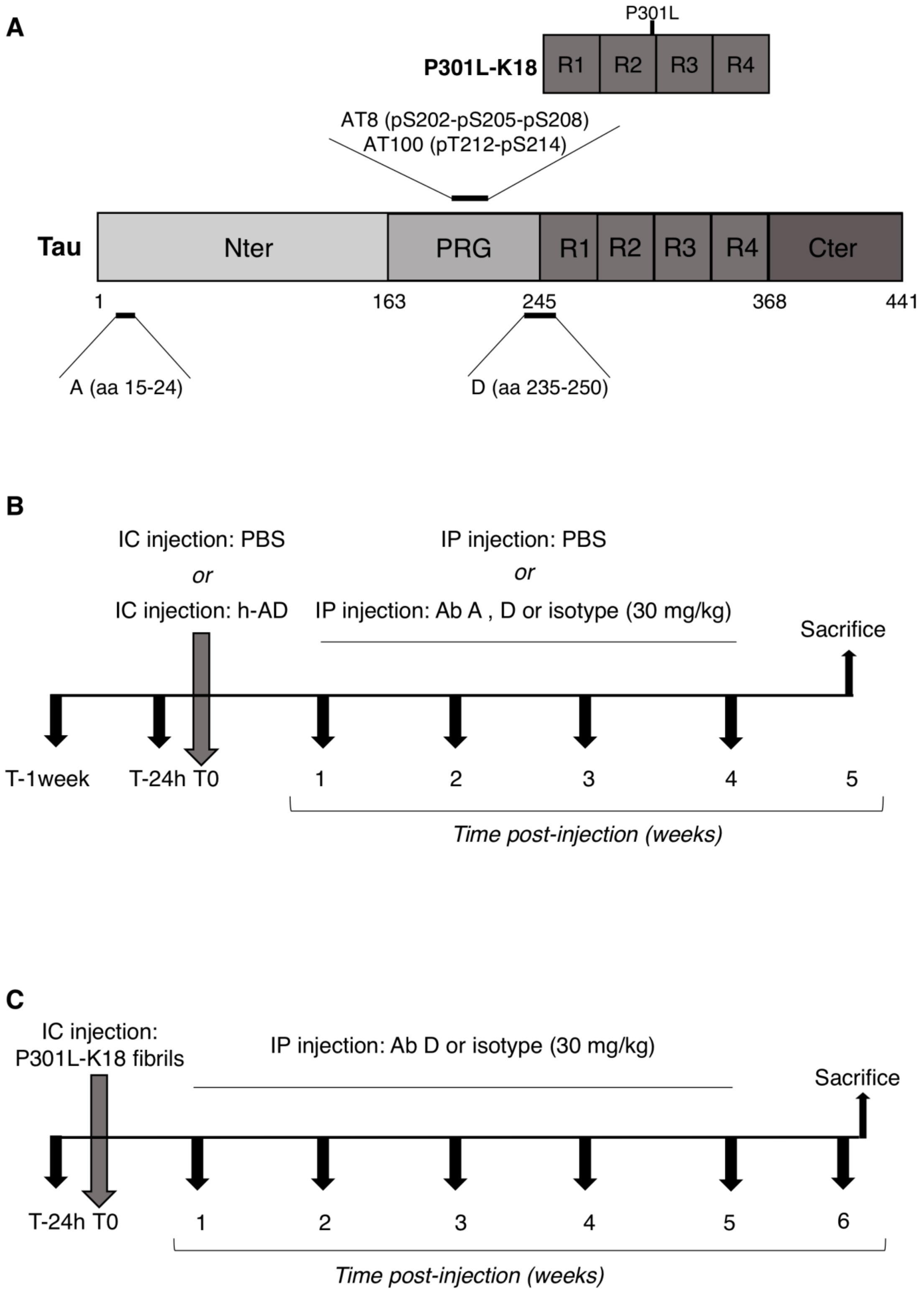

Supplementary figure 1- Study design of passive immunization in Tg30tau and in htauP301L tg mice. (**A**) Abs epitopes- AT8 (pS202, pT205, pS208) (Malia *et al.*, 2016), AT100 (pT212-pS214); Ab A targets the N-terminal part of tau (amino acids 15 to 24); Ab D binds a central epitope close to the 4 microtubules binding domains (amino acids 235 to 250). P301L mutation in the K18 sequence is shown in the upper panel. (**B**) Intracranial (IC) injections of PBS (2 µl) or h-AD (2 µl, 5.5 µg/µl) were performed in the right CA1 layer of 1-month-old Tg30tau or littermate mice. Animals received intraperitoneal (IP)

administration of PBS or Abs A, D or isotype at 30 mg/kg 1 week and 24 h prior to human h-AD or PBS injection in the brain. Immunizations were performed weekly up to sacrifice 5 week p.i.. (**C**) 4 months-old htauP301L tg mice were immunized (D or isotype Abs, 30 mg/kg) 24 h prior to P301L-K18 fibrils (1 µl, 5 µg/µl) or PBS (1 µl) injection in the right hippocampus. Immunization was maintained once a week until sacrifice 6 week p.i. and the mice were sacrificed 24 h later.

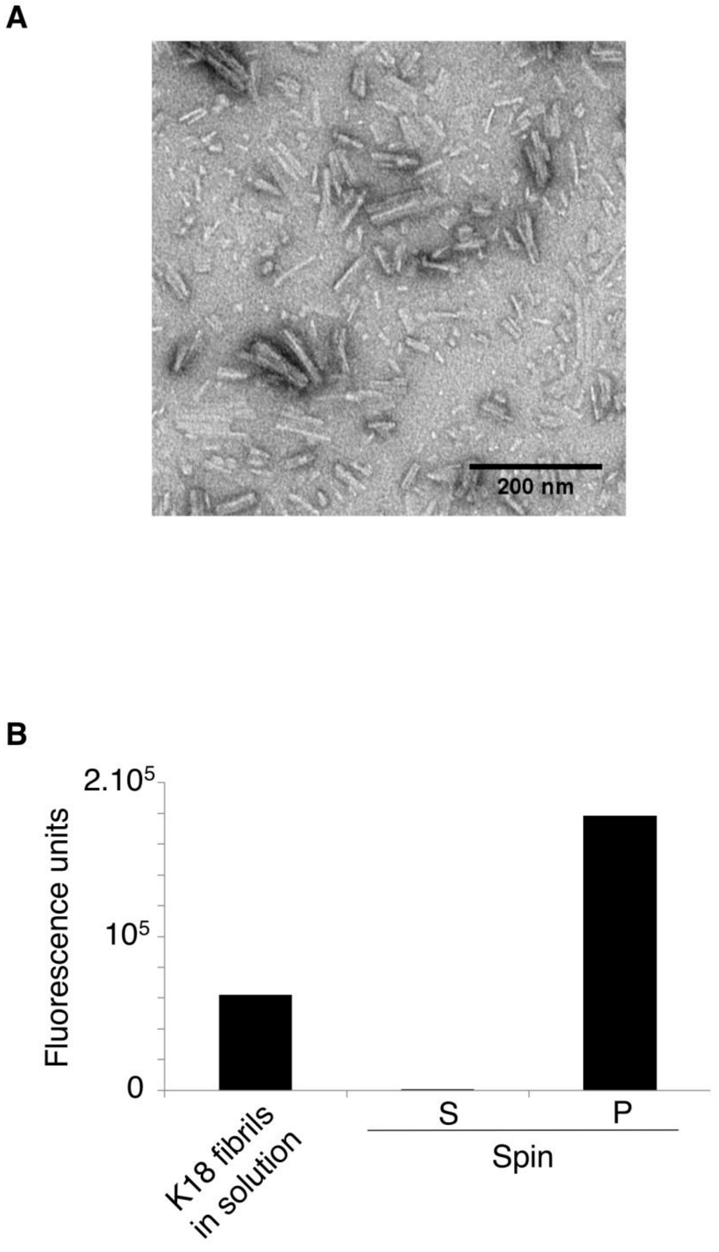

Supplementary figure 2- K18 fibrils characterization. **(A)** Electron micrograph of K18 fibrils after negative staining, scale bar is indicated on the figure, **(B)** Thioflavine T incorporation into K18 fibrils in the original sample, the supernatant (S) and the pellet (P) fractions generated after centrifugation (100,000g).

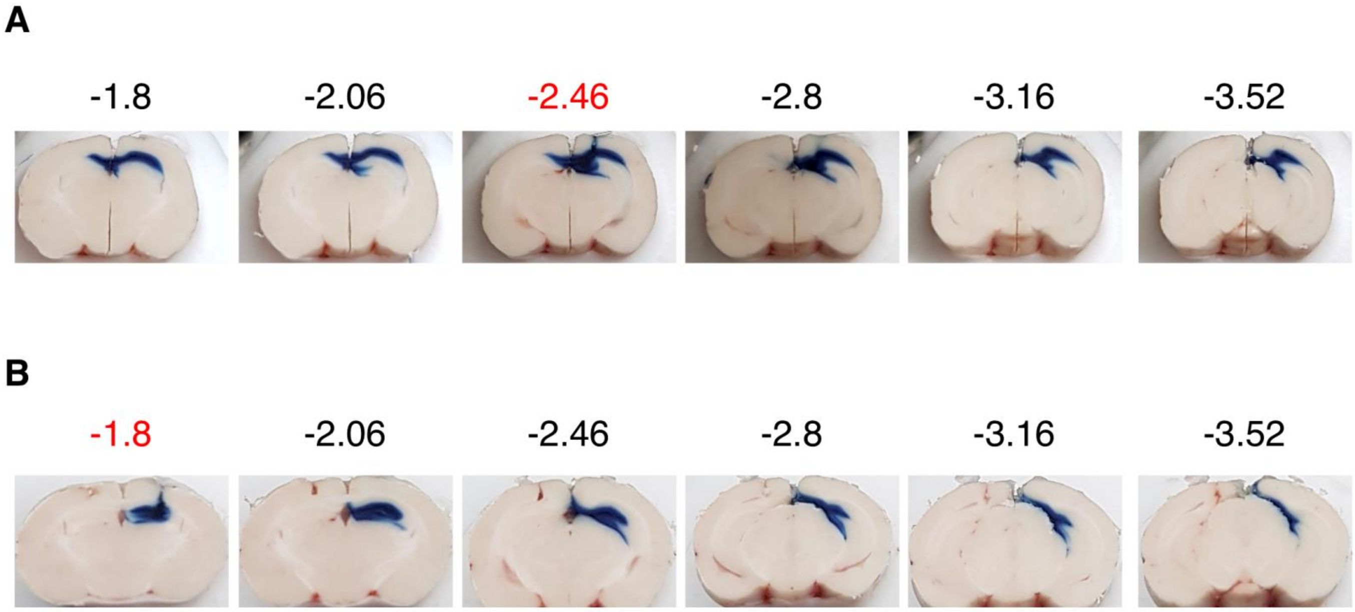

Supplementary figure 3- Targeting of hippocampus. Blue Evans (1%) were injected in the right hippocampus either of 1-month-old Tg30tau mice (**A**) (n=2, Anterior-Posterior: -2.5 mm; Medial-Lateral: -1 mm; Dorsal-Ventral: -1.8 mm to bregma) or 4 months-old htauP301L tg (**B**) (n=2, Anterior-Posterior: -1.8 mm; Medial-Lateral: -1.72 mm; Dorsal-Ventral: -1.8 mm). After sacrifice, the brains were removed and free-floating coronal cryostat sections were done to analyse the blue Evans diffusion. Photographs of six brain sections covering the entire hippocampus were shown (Bregma -1.7 to -3.52). In **A** and **B**, injection sites are shown in red.

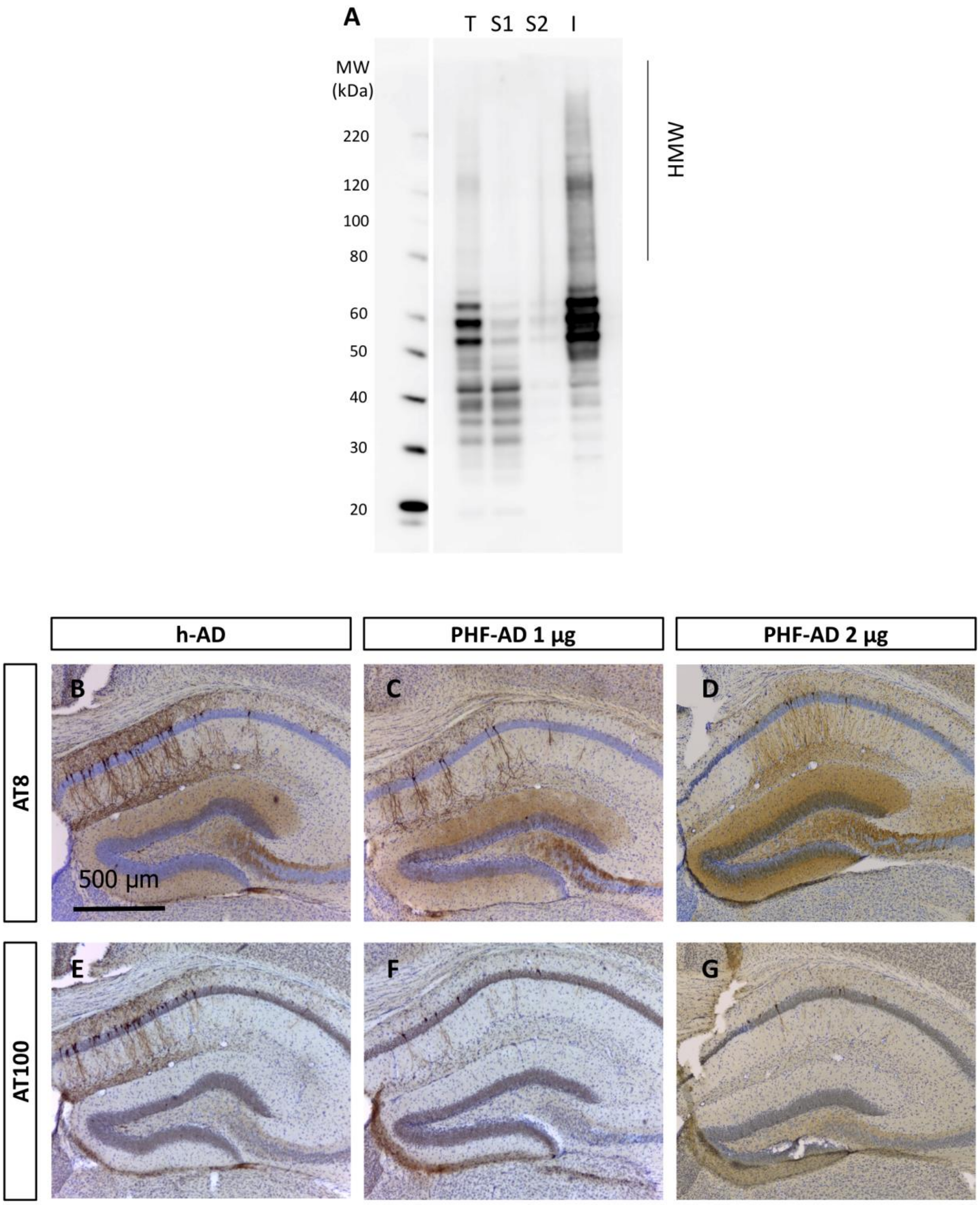

Supplementary figure 4- Human Alzheimer's disease brain homogenate and human purified PHF seeding activity. (**A**) Alzheimer's disease brain extract was processed for western-blot after sarkosyl soluble and insoluble extractions using M19G Ab. T=total homogenate, S1=triton-soluble fraction, S2=sarkosyl-soluble fraction, I=sarkosyl-insoluble fraction. HMW tau species are indicated on the blot. h-AD (**B, E**) (2 µl, 5.5 µg/µl, n=2 mice) or human Alzheimer’s disease purified PHF (**C, D, F, G**) (1 or 2 µg, n=2 mice per group) were unilaterally injected in 1-month-old Tg30tau mice. 5 weeks p.i., animals were sacrificed and DAB-immunostaining were performed with AT8 (**B-D**) or AT100 (**E-G**) Abs at the injection site in the ipsilateral hippocampus.

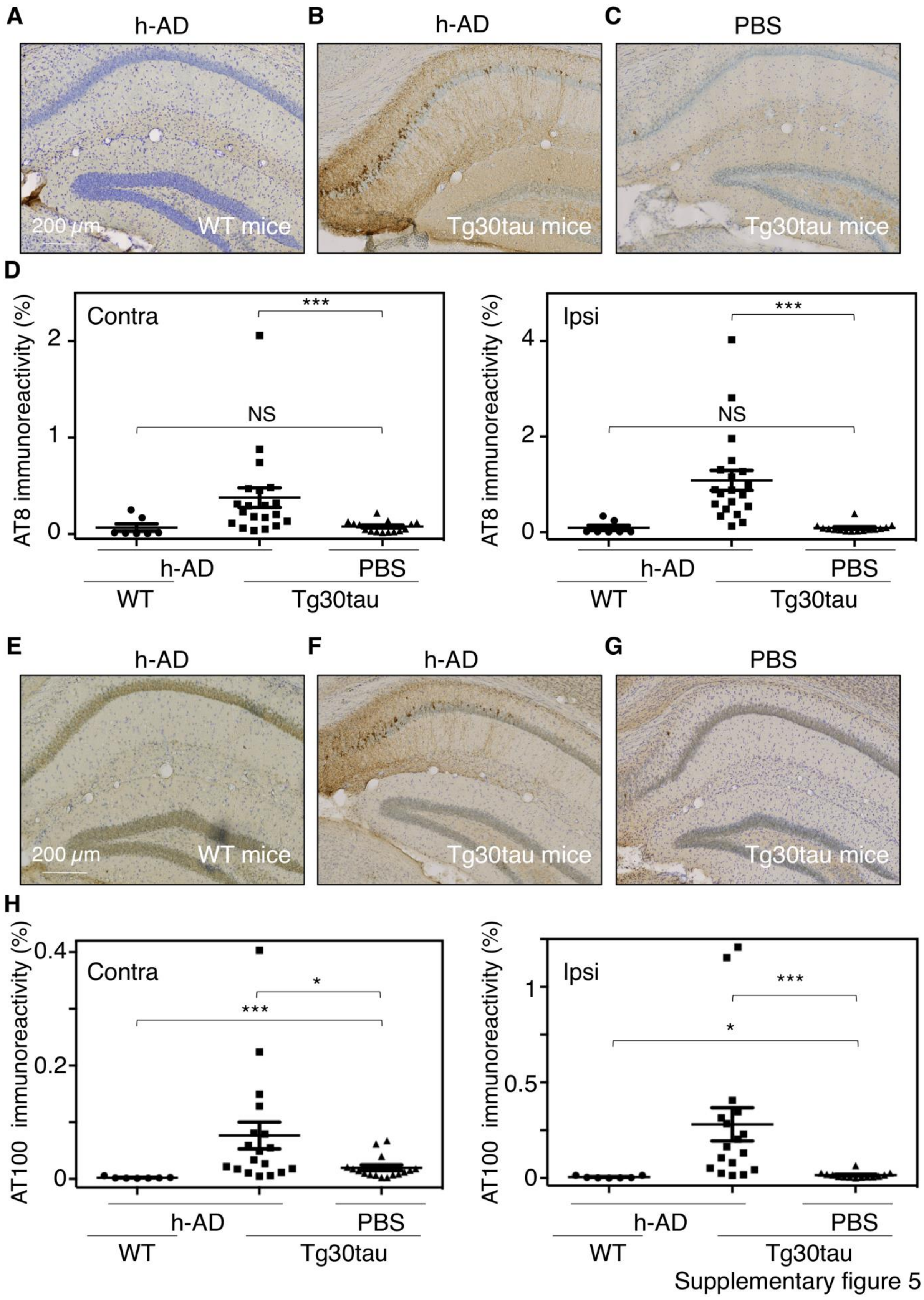

Supplementary figure 5- Alzheimer's disease brain lysate seeds human mutated tau expressed in Tg30tau mice. Alzheimer’s disease brain lysate (h-AD, 2 µl, 5.5 µg/µl) or PBS (2 µl) was unilaterally injected in 1-month-old Tg30tau mice. 5 weeks p.i., animals were killed and DAB-immunostaining was performed with AT8 or AT100 Abs. AT8 immunoreactivity at the injection site of WT mice (**A**), Tg30tau mice (**B**) injected with Alzheimer's disease brain lysate or Tg30tau mice injected with PBS (**C**) is shown.

Percentage of AT8 immunopositive areas in the ipsi- and contralateral CA1 layer (**D,** Tg30tau mice injected with h-AD versus Tg30tau mice injected with PBS: in the ipsilateral side, Z=-4.78, p<0.0001 and in the contralateral side, Z=-3.65, p=0.0003-Littermate wild-type mice injected with h-AD versus Tg30tau mice injected with PBS: ipsilateral side, Z=-1.69, p=0.0907 and contralateral side, Z=-1.62, p=0.105). AT100 immunoreactivity at the injection site of WT mice (**E**), Tg30tau mice (**F**) injected with h-AD or Tg30tau mice injected with PBS (**G**) is shown. Percentage of AT100 immunopositive areas in the ipsi- and contralateral CA1 layer (**H,** Tg30tau mice injected with h-AD versus Tg30tau mice injected with PBS: in the ipsilateral side, Z=-4.10, p<0.0001 and in the contralateral side, Z=-2.46, p=0.0136- Littermate wild-type mice injected with h-AD versus Tg30tau mice injected with PBS: ipsilateral side, Z=-2.20, p=0.0277 and contralateral side, Z=-3.5, p=0.0005). For **D** and **H**, six brain sections covering the entire hippocampus were quantified (Bregma -1.7 to -3.52). Data are presented as mean ± SEM and analyzed by Mann & Whitney tests (n =7 WT mice+h-AD, n=15 Tg30tau mice+h-AD, n=13 Tg30tau mice+PBS). *p < 0.05, *** p < 0.001.

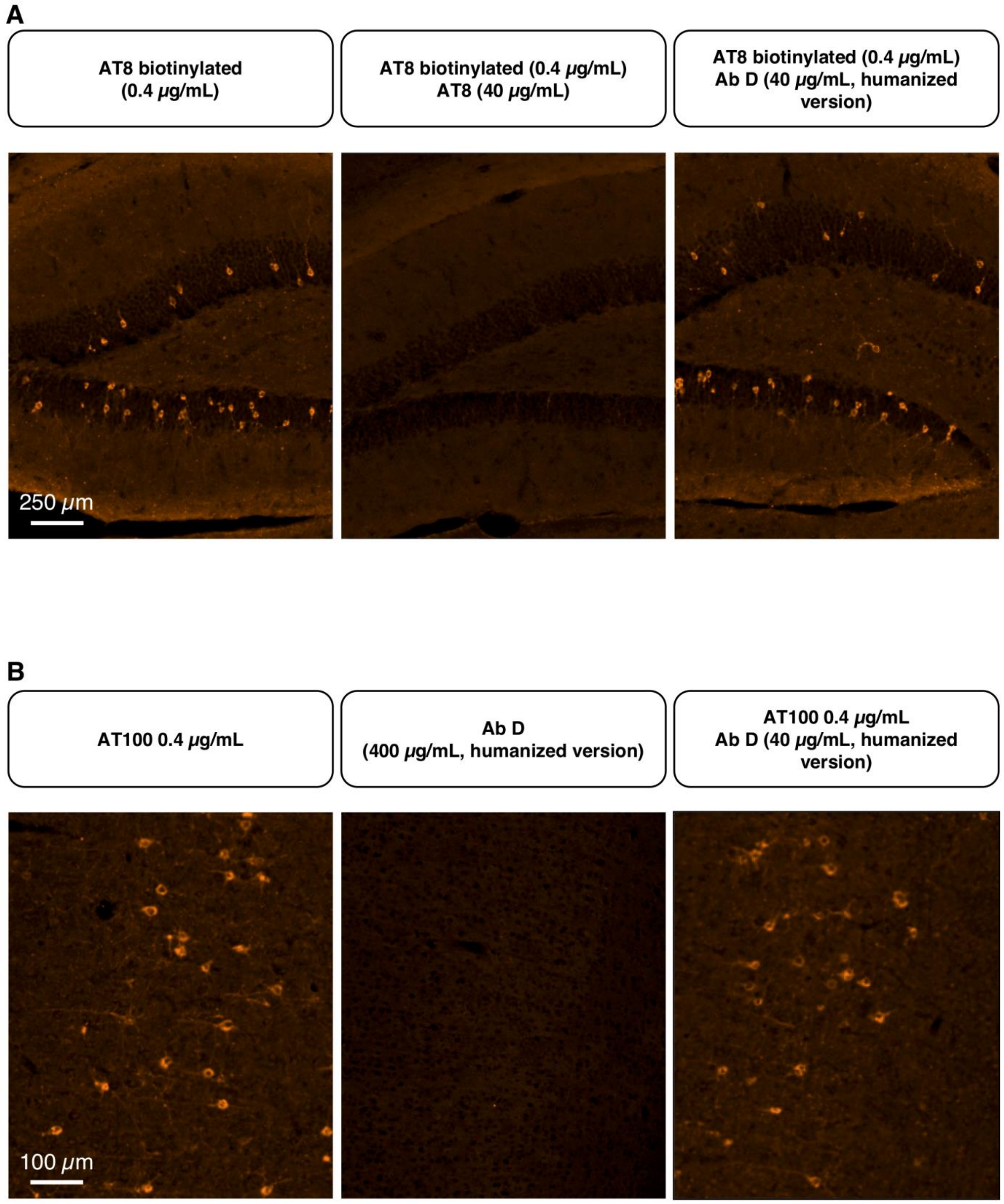

Supplementary figure 6- Competition assays. Tau lesions were detected in hippocampal (**A**) or cortical (**B**) brain sections from 5.5 months old htauP301L mice using primary biotinylated AT8 Ab (0.4 µg/ml) with (**A**, right part) or without (**A**, left part) co-incubation period of slices with the human version of Ab D (40 µg/ml). Primary Ab was revealed using secondary Alexa Fluor 546 streptavidin anti-mouse Ab. The same was done to reveal AT100 immunoreactivity (0.4 µg/ml) with (**B**, right part) or without (**B**, left part) pre-incubation of slices with the human version of Ab D (40 µg/ml). Primary Ab was detected using Alexa 546 polyclonal goat anti-mouse Ab.

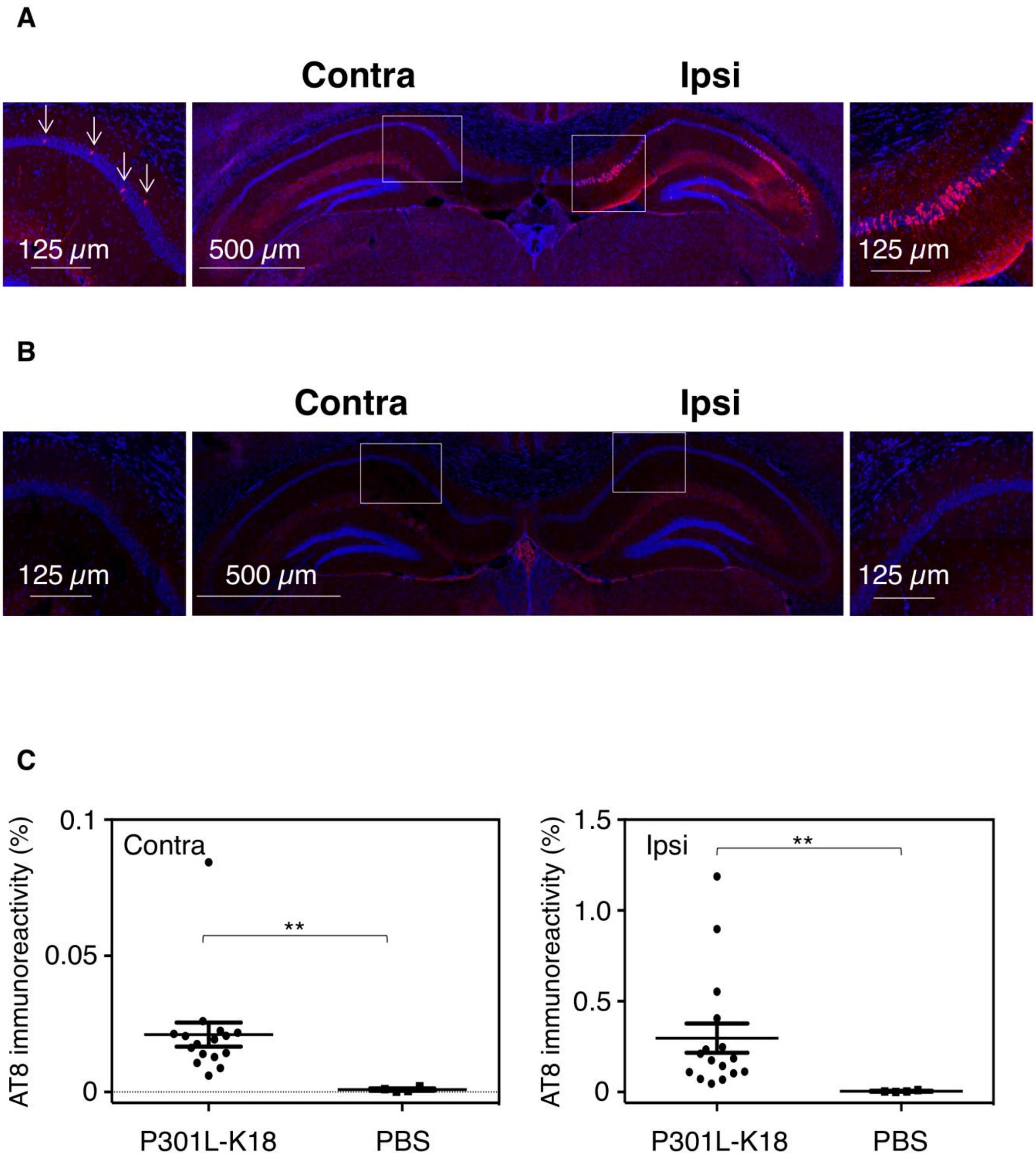

Supplementary figure 7- Recombinant P301L-K18 fibrils seed human mutated tau expressed in htauP301L tg mice. P301L-K18 fibrils (1µl, 5 µg/µl) (**A**) or PBS (1 µl) (**B**) were unilaterally injected in 4 months-old htauP301L tg mice. 6 weeks later, animals were sacrificed and immunofluorescent staining performed using AT8 Ab. Sections from the hippocampus (injection bregma) are shown. (**C**) Percentage of AT8 immunopositive areas in the ipsi- and contralateral hippocampus. For **C**, twenty brain sections (Bregma -2.06. to -3.64 mm) were quantified. Data are presented as mean ± SEM and analysed by Mann & Whitney tests (n=15 hP301L Tg mice+P301L-K18, n=4 hP301L Tg mice+PBS, Z=-2.95, p=0.0032 for the ipsilateral side and Z=-2.97, p=0.0029 for the contralateral side, ** p < 0.01).

A

Antibody	Reactivity	Species	Supplier	Reference	Dilution (blocking)
**Immunohistochemistry**					
**AT8**	Human tau pS202/pT205/pS208	Mouse	Thermofisher	MN1020	1:500
**AT100**	Human tau pT212/pS214	Mouse	Thermofisher	MN1060	1:1,000
**AT8 biotinylated**	Human tau pS202/pT205/pS208	Mouse	Thermofisher	MN1020B	1:30,000
**Western blot**					
**M19G**	Human tau Nter domain	Rabbit	Home-made (Buée's lab)	n.a.	1:10,000 (5% milk)
**pS396**	Human tau pS396	Rabbit	Invitrogen	44-752G	1:10,000 (5% BSA)
**AT100**	Human tau pT212/pS214	Mouse	Thermofisher	MN1060	1:500 (w/o blocking)
**Ab A**	Human tau Nter domain	Mouse	UCB Biopharma	n.a.	1:2,500 (5% BSA)
**Ab D**	Human tau 235-250 aa	Mouse	UCB Biopharma	n.a.	1:2,500 (5% BSA)
**Isotype**	human TNFα	Mouse	UCB Biopharma	n.a.	1:2,500 (5% BSA)
**Immunofluorescence**					
**AT8 biotinylated**	Human tau pS202/pT205/pS208	Mouse	Thermofisher	MN1020B	1:500
**AT100**	Human tau pT212/pS214	Mouse	Thermofisher	MN1060	1:500

B

Antibody	Selectivity	Tau monomer			AD-PHF		
		ka (1/Ms)x10^5	kd (1/s)x10^5	kD (nM)	ka (1/Ms)x10^5	kd (1/s)x10^5	kD (nM)
**Ab A**	Total Tau	3.6	5.2	0.16	2.3	2.5	0.12
**Ab D**	Total Tau	2.1	25.3	1.2	1.4	10.3	0.8

Table 1- Abs used in the study. (A) For immunohistochemistry/immunofluorescence and biochemical assays and **(B)** Characterization of anti-Tau Abs by surface plasmon resonance. Binding parameters were determined for anti-tau Abs against monomeric tau and PHF Tau isolated from Alzheimer's disease brain pools. Abs A and D display similar binding profiles against monomeric and PHF Tau but Ab A has a 7-fold better affinity than Ab D.

Full length blots

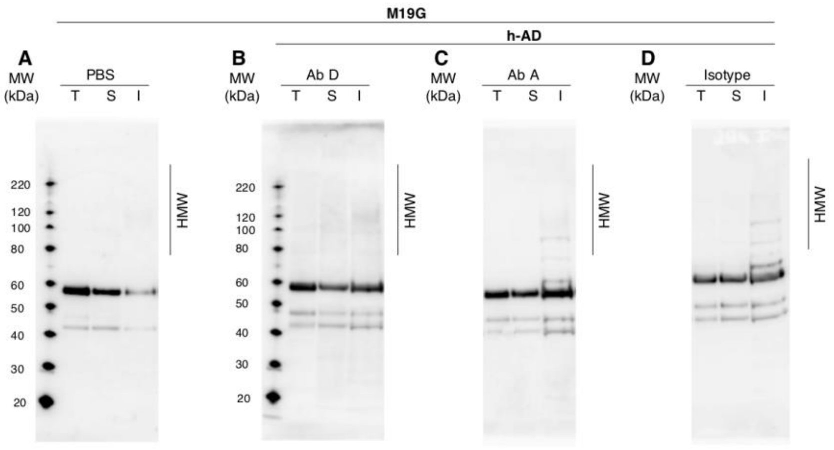

Reference

Malia TJ, Teplyakov A, Ernst R, Wu SJ, Lacy ER, Liu X, et al. Epitope mapping and structural basis for the recognition of phosphorylated tau by the anti-tau antibody AT8. Proteins 2016; 84:427–34.
